# Downregulation of the PD-1/PD-Ls pathway in peripheral cells correlates with asbestosis severity

**DOI:** 10.1186/s12890-021-01531-5

**Published:** 2021-05-22

**Authors:** Meihua Qiu, Yuqing Chen, Qiao Ye

**Affiliations:** 1 Department of Occupational Medicine and Toxicology, Beijing Institute of Respiratory Medicine, Beijing Chao-Yang Hospital, Capital Medical University, No. 8 Worker’s Stadium, Chao-Yang District, Beijing, China; 2 Department of Respiratory and Critical Care Medicine, Yantai Yuhuangding Hospital, Affiliated with the Medical College of Qingdao, Yantai, Shandong China; 3 Department of Respiratory and Critical Care Medicine, The Fifth Hospital of Xiamen, Xiamen, Fujian China

**Keywords:** Asbestosis, Silicosis, Lung fibrosis, PD-1, PD-Ls, Lung function, T cell activation

## Abstract

**Background:**

Asbestosis and silicosis are characterized by diffuse or nodular interstitial lung fibrosis resulting from exposure to asbestos or silica dust, respectively. This study was designed to detect programmed cell death protein (PD-1)/programmed death ligands (PD-Ls) expression in patients with asbestosis and silicosis and to explore the possible clinical significance of PD-1/PD-Ls expression in patients with the two diseases.

**Methods:**

Thirty patients with asbestosis, 23 patients with silicosis and 25 healthy controls were consecutively recruited and provided informed consent to participate in the study. Clinical data were collected from patients’ clinical charts. PD-1/PD-Ls expression in peripheral blood (PB) was detected using flow cytometry.

**Results:**

PD-1 was expressed at significantly lower levels on CD4^+^ or CD8^+^ peripheral T cells from patients with asbestosis and silicosis than on cells from healthy controls. Similarly, significantly lower PD-L1 and PD-L2 expression was detected on CD14^+^ monocytes from patients with asbestosis and silicosis than on cells from healthy controls. In addition, no significant differences in PD-1, PD-L1 and PD-L2 expression were observed between the asbestosis and silicosis groups. Moreover, the proportions of PD-1^+^ CD4^+^ T cells and PD-1^+^ CD8^+^ T cells in patients with asbestosis were positively correlated with the percentage of forced vital capacity predicted.

**Conclusions:**

Decreased PD-1 expression on CD4^+^ T or CD8^+^ T cells in PB was positively correlated with the asbestosis severity, implying that pulmonary fibrosis development in patients with asbestosis was positively correlated with the downregulation of the PD-1/PD-Ls pathway.

**Supplementary Information:**

The online version contains supplementary material available at 10.1186/s12890-021-01531-5.

## Background

Interstitial lung disease (ILD), or diffuse parenchymal lung disease, is a group of diseases that appear in the lung interstitium and alveolar cavity, resulting in alveolar capillary dysfunction [1]. Pulmonary fibrosis is an advanced histopathological feature of various ILDs. Pneumoconiosis, an ILD associated with occupational environments, is a heterogeneous group of diseases caused by inorganic mineral dust. Exposure to occupational dust can induce a cascade of lung inflammation and structural damage that potentially leads to dust-related lung disorders, including pneumoconiosis and chronic obstructive pulmonary disease (COPD) [2]. As shown in our previous study, occupational dust exposure and heavy smoking are associated with an increased risk of combined COPD and pneumoconiosis, especially in patients with silicosis and coal workers’ pneumoconiosis [3].

Asbestosis and silicosis are two types of pneumoconiosis. Asbestosis is characterized by diffuse interstitial pulmonary fibrosis caused by long-term asbestos exposure. The most common clinical symptom of asbestosis is progressive dyspnoea on exertion. The disease is associated with a restrictive lung impairment and decreased diffusing capacity [4]. Chest high-resolution computed tomography (HRCT) mainly shows usual interstitial pneumonia (UIP) or nonspecific interstitial pneumonia (NSIP) [1]. Silicosis is a nodular fibrotic lung disease caused by the inhalation of free crystalline silicon dioxide or silica and is also one of the most important occupational diseases worldwide [5]. In our recent study, we reported an outbreak of accelerated silicosis caused by artificial stone dust [6]. Artificial stone-associated silicosis is characterized by a shorter latency, rapid radiological progression and accelerated loss of lung function, which differs from natural stone-associated silicosis [6].

Asbestosis and silicosis are currently incurable and may be progressive even after dust exposure cessation [7]. The pathogenesis of asbestosis and silicosis remains unclear. Therefore, studies aiming to obtain a deeper understanding of the mechanisms underlying the development of asbestosis and silicosis and to identify potential targets for the treatment of the diseases will provide a foundation for the development of new therapies.

Programmed cell death protein 1 (PD-1), also known as CD279, is a member of the B7/CD28 immunoglobulin superfamily. PD-1 has two ligands, programmed death ligand-1 (PD-L1 or CD274) and programmed death ligand-2 (PD-L2 or CD273) [8]. In the process of antigen presentation, PD-1 interacts with PD-L1 or PD-L2 and then inhibits T cell activation and reduces cytokine production. Thus, PD-1 functions as a negative regulator of this process [9].

No existing study has shown whether the PD-1/PD-Ls signalling pathways play a role in cellular immunology in pulmonary fibrotic diseases caused by asbestos or silica. We explored the expression of members of the PD-1/PD-Ls pathway in patients with asbestosis and silicosis and further elucidated the relationship between the expression of these molecules and clinical indexes. Here, we described the expression of components of the PD-1/PD-Ls pathway in human pulmonary fibrosis, which provided a deeper understanding of the mechanism underlying the development of pulmonary fibrosis and might provide clues for the identification of new drug targets for the treatment of pulmonary fibrosis. Our study found that decreased PD-1 expression on CD4^+^ or CD8^+^ T cells in peripheral blood (PB) was positively correlated with the disease severity of asbestosis.

## Methods

### Patients and control subjects

Thirty patients with asbestosis and 23 patients with silicosis were consecutively recruited from the Department of Occupational Medicine and Toxicology, Beijing Chao-Yang Hospital. Clinical data, including age, sex, current and past medical history, occupational history and pulmonary function values, were collected from patients’ clinical charts.

Asbestosis was diagnosed in patients based on the criteria listed below. (1) Patients had a definite occupational history of exposure to chrysotile dust through asbestos product manufacturing and a prolonged latency. Chrysotile asbestos is a general term for serpentine asbestos. Serpentine asbestos is a trioctahedral silicate mineral with a double-layer structure composed of SiO_2_ tetrahedra and Mg(OH)_2_ octahedra. (2) Patients’ lungs exhibited honeycombing and septal and interlobular fissure thickening, as well as diffuse pleural thickening and/or pleural plaques, on chest HRCT; and (3) patients in whom other known causes of ILD were excluded [10]. Silicosis was diagnosed in patients based on the following criteria: (1) patients with a definite occupational history of exposure to silica dust through stone sculpture manufacturing and a prolonged latency; (2) patients whose lungs featured multiple small nodules along the lymphatic distribution, as well as nodules that even fused into masses, on chest HRCT; and (3) patients in whom tuberculosis and malignancy were excluded [11]. Twenty-five subjects who underwent routine health examinations and showed no evidence of disease were enrolled in the study as healthy controls.

All patients were in a stable clinical state and showed no clinical, radiographic, or electrocardiographic signs of heart failure, acute pulmonary infection, or pulmonary thromboembolism. None of the patients were receiving treatment with corticosteroids and/or immunosuppressants. The study protocol (No. 13-KE-61) was approved by the Ethics Committee of Beijing Chao-Yang Hospital, Capital Medical University. All the participants provided written informed consent before enrolling in the study.

### Computed tomography scans

HRCT was performed with 1-mm sections and 1-s scan times. The apex-base scans, which included both lungs in the field of view, were performed at 10-mm intervals. The CT images were reviewed independently by two experienced thoracic radiologists, and the CT patterns were obtained and recorded by two observers (MHQ and YQ). Patients who showed evidence of coexisting emphysema (> 5% of total lung volume) on HRCT were not included in the study.

### Pulmonary function tests

Pulmonary function tests were performed according to American Thoracic Society guidelines [12]. We recorded the arterial partial pressure of oxygen (PaO_2_), forced vital capacity (FVC), forced expiratory volume in the first second (FEV_1_), FEV_1_/FVC ratio, total lung capacity (TLC), and diffusing capacity of the lung for carbon monoxide (DLCO) (single-breath method, with the values corrected for the current haemoglobin level) of each subject.

### Cell collection

PB samples were collected from each subject, placed in ethylenediaminetetraacetic acid-treated tubes and processed to measure PB mononuclear cell (PBMC) counts for subsequent flow cytometry procedures. The blood samples were layered onto Ficoll-Paque Plus (Amersham Biosciences, Amersham, Bucks, UK), centrifuged (400 g for 20 min at 21 °C), and then PBMCs were harvested. The cells were washed once with divalent cation-free Hanks balanced salt solution at 300 g for 5 min at 4 °C. The PBMCs were subsequently resuspended, and the number of viable cells was counted.

### Flow cytometry

Freshly obtained human PBMC samples were stained with anti-hCD273-FITC, anti-hCD279-PE, anti-hCD4-APC, anti-hCD8-PerCP, anti-hCD14-FITC, anti-hCD274-PE, anti-hCD45-PerCP and matched isotype control antibodies and incubated in a dark room for 30 min at 4 °C. Anti-hCD8-FITC, anti-hCD4-APC, anti-hCD28-PE, anti-hCD69-PerCP, anti-hHLA-DR-PerCP, and anti-hCD38-PE antibodies were used for surface marker staining of effector T cells. All antibodies were purchased from BD Biosciences (San Jose, CA, USA). Data acquisition and analysis were performed with Canto II Software (BD Biosciences, San Jose, CA, USA). Approximately 10^5^ cells were acquired for subsequent data analyses.

### Statistical analysis

Data are presented as the means ± standard deviations (SD) or as medians and interquartile ranges (IQRs) when appropriate. Group comparisons were performed using analysis of variance, Student’s *t* test, Wilcoxon’s rank-sum test, or the chi-square test, as appropriate, and correlations were assessed by calculating Pearson’s correlation coefficient or Spearman’s rank correlation coefficient. *P* < 0.05 was considered statistically significant. Statistical analyses were performed with SPSS for Windows V17.0 (Chicago, IL, USA) and GraphPad Prism 5 software (San Diego, CA, USA).

## Results

### Demographic characteristics of the study population

Thirty patients with asbestosis, 23 patients with silicosis and 25 healthy controls were evaluated in this study (Table 1). None of the participants enrolled in the study were current smokers. Patients with silicosis and healthy controls were significantly younger than patients with asbestosis (*P* < 0.01); however, no significant difference in age was observed between the silicosis and healthy control groups. The PaO_2_ level was significantly lower in the asbestosis group than in the silicosis and control groups (*P* < 0.05). Pulmonary function tests indicated that restrictive ventilation and/or impaired gas exchange were present in the asbestosis group, while normal lung function or mild airflow limitation and a mildly decreased DLCO were present in the silicosis group.

**Table 1:** Demographics of asbestosis, silicosis and healthy controls

	Asbestosis	Silicosis	Controls	*P* value*
Subjects	30	23	25	–
Age, years	71.3 ± 8.7	52.9 ± 12.0	46.8 ± 5.4	0.000
Female/male, n	17/13	14/9	7/18	0.040
Smoker/non-smoker,n	12/18	4/19	13/12	0.043
PaO_2_, mmHg	77.63 ± 11.73	79.94 ± 14.12	87.92 ± 2.69	0.041
FVC, %pred	61.18 ± 23.30	88.87 ± 22.36	89.16 ± 2. 54	0.000
FEV_1_, %pred	59.74 ± 24.15	70.47 ± 27.59	89.80 ± 8.45	0.000
FEV_1_/FVC, %	75.81 ± 10.90	67.07 ± 16.55	87.24 ± 2.82	0.000
TLC, %pred	74.07 ± 20.87	83.02 ± 31.25	90.52 ± 3.02	0.290
DLCO, %pred	55.86 ± 25.93	76.47 ± 17.96	85.84 ± 3.24	0.000

Data are presented as Means ± SD.*: *P* value denotes statistical differences among three groups; PaO_2_, the arterial partial pressure of oxygen; FVC, forced vital capacity; FEV_1_, forced expiratory volume in the first second; TLC, total lung capacity; DLCO, diffusing capacity of the lung for carbon monoxide

### ***PD-1 expression on circulating CD4***^+^***or CD8***^+^***T cells was decreased in patients with asbestosis or silicosis***

**Fig. 1:**
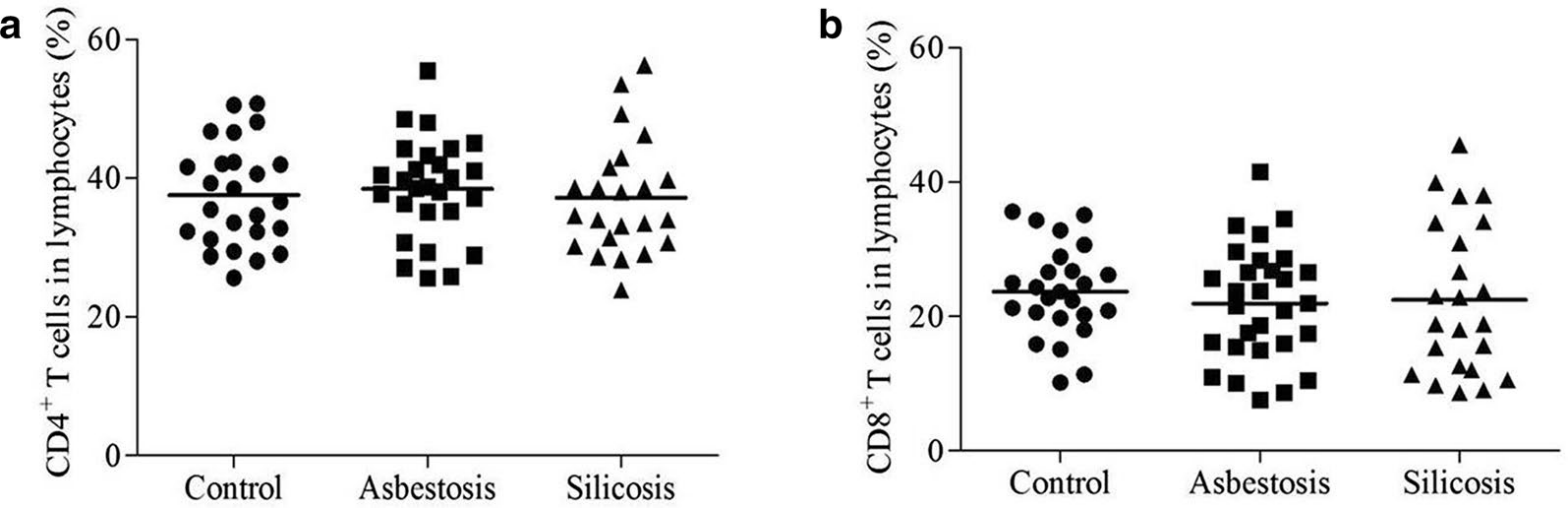
Percentages of CD4^+^ or CD8^+^ T cells among lymphocytes in PB. The percentages of CD4^+^ (**a**) or CD8^+^ (**b**) T cells among lymphocytes in the PB of healthy controls and patients with asbestosis or silicosis

**Fig. 2:**
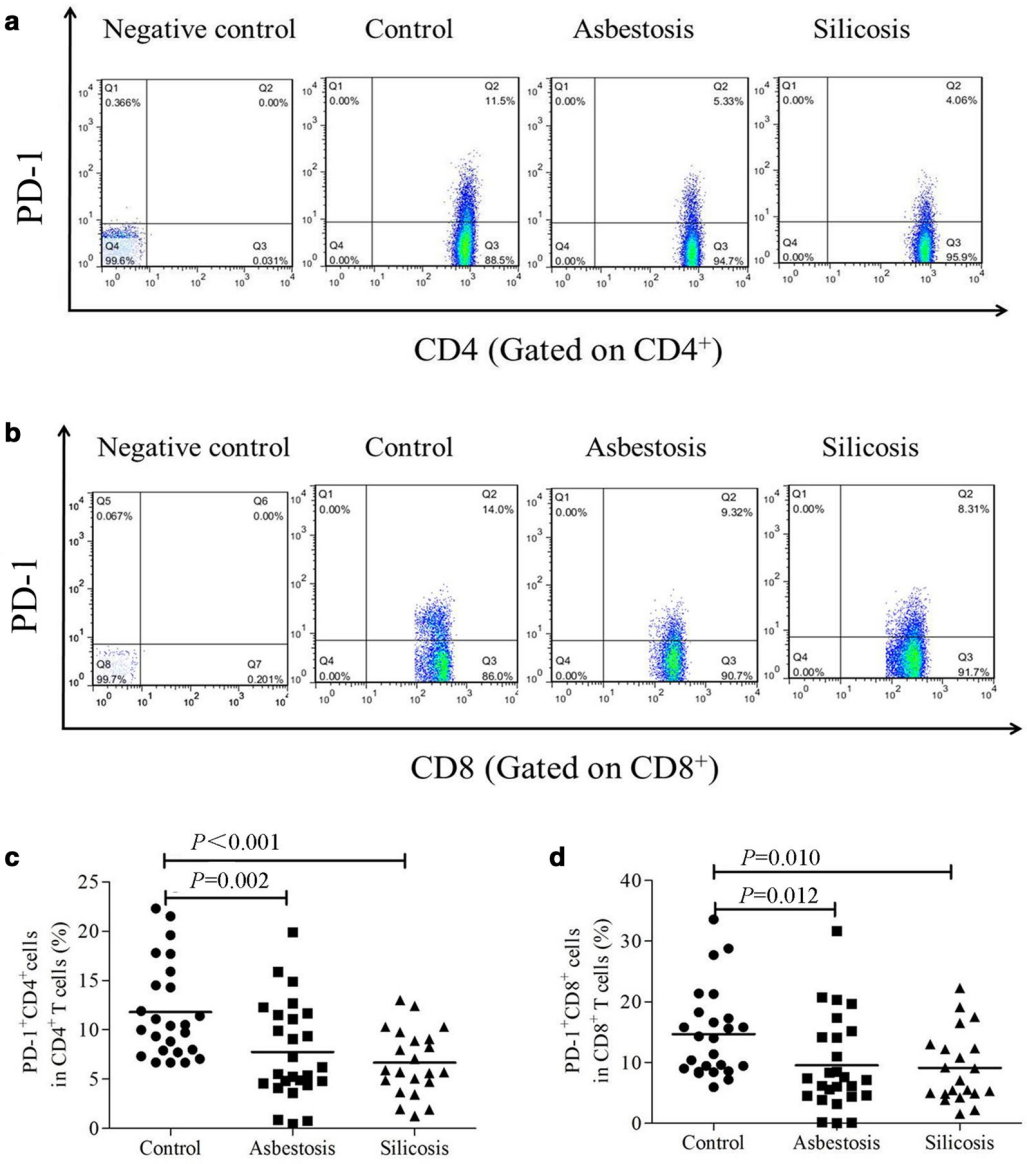
Analysis of PD-1 expression on CD4^+^ T or CD8^+^ T cells in PB. Representative flow cytometry analysis of the expression of PD-1 on CD4^+^ (**a**) or CD8^+^ (**b**) T cells. The percentages of PD-1^+^CD4^+^ (**c**) or PD-1^+^CD8^+^ (**d**) T cells in healthy controls and patients with asbestosis or silicosis (**c**, **d**)

Figure 1 shows no significant differences in circulating CD4^+^ or CD8^+^ T cell fractions among the three groups (*P* > 0.05). We first investigated PD-1 (CD279) expression in PB. As shown in Fig. 2a, c, significantly lower PD-1 expression was detected on CD4^+^ T cells in PB from the asbestosis (mean 7.753%) and silicosis (mean 6.676%) groups than in PB from the healthy control group (mean 11.790%, *P* < 0.01). Similarly, PD-1 expression on CD8^+^ T cells in PB was also significantly decreased in the asbestosis (mean 9.556%) and silicosis groups (mean 9.132%) compared to the healthy control group (mean 14.670%, *P* < 0.05) (Fig. 2b, d). However, a significant difference in PD-1 expression was not observed between the asbestosis and silicosis groups (Fig. 2c, d).

### ***PD-L1 expression on circulating CD4***^+^***T cells was decreased in patients with asbestosis or silicosis***

**Fig. 3:**
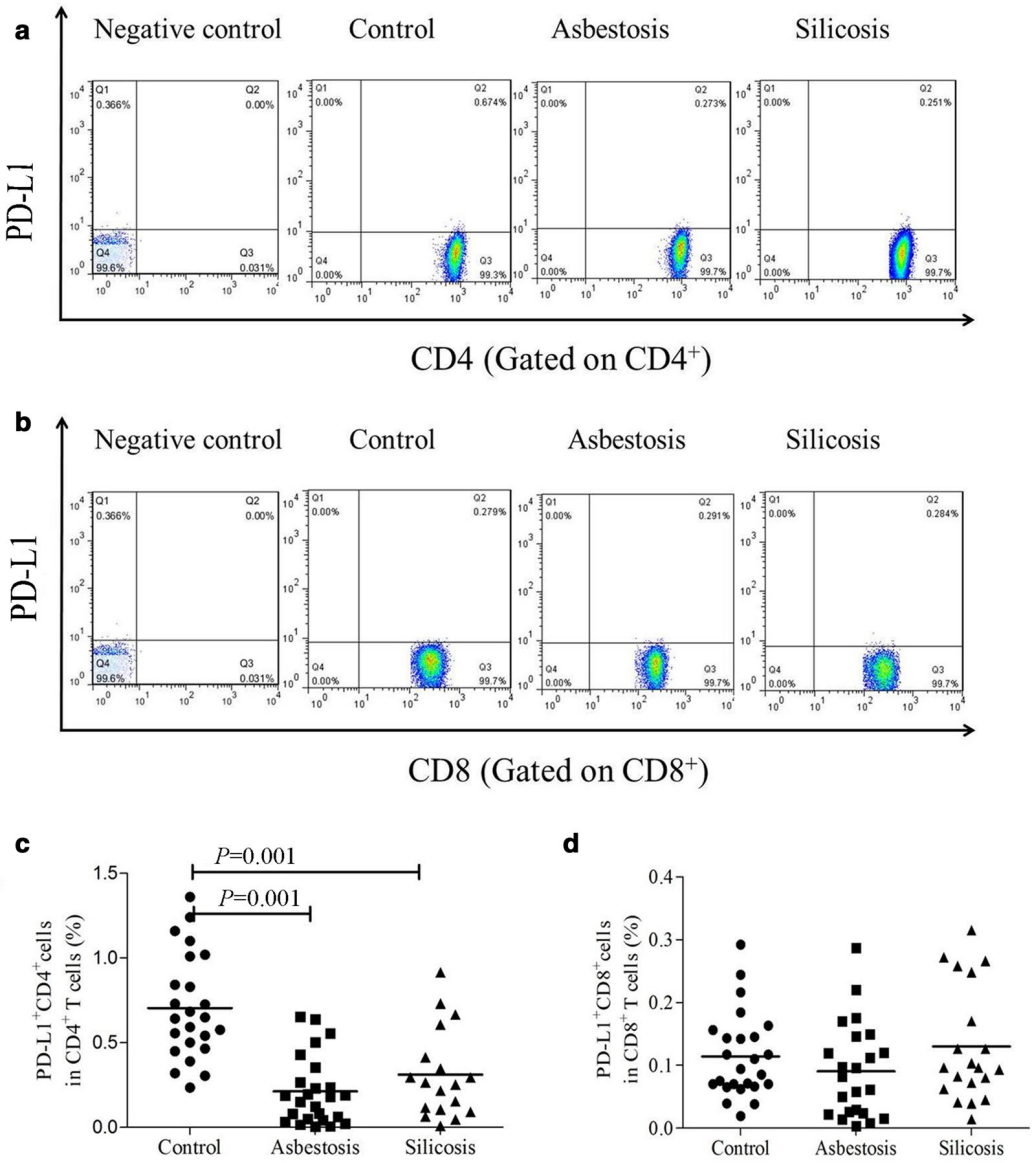
Analysis of PD-L1 expression on CD4^+^ T or CD8^+^ T cells in PB. Representative flow cytometry analysis of the expression of PD-L1 on CD4^+^ (**a**) or CD8^+^ (**b**) T cells. The percentages of PD-L1^+^CD4^+^ (**c**) or PD-L1^+^CD8^+^ (**d**) T cells in healthy controls and patients with asbestosis or silicosis (**c**, **d**)

As shown in Fig. 3, PD-L1 (CD274) was mainly detected on circulating CD4^+^ T cells rather than on CD8^+^ T cells in the healthy controls. However, PD-L1 expression on circulating CD4^+^ T cells was significantly decreased in the asbestosis (mean 0.212%) and silicosis (mean 0.310%) groups compared to the healthy control group (mean 0.705%) (*P* < 0.05) (Fig. 3a, c). No significant difference in PD-L1 expression on circulating CD8^+^ T cells was observed among the three groups (Fig. 3b, d).

### ***Decreased PD-L1 and PD-L2 expression on circulating CD14***^+^***monocytes in patients with asbestosis or silicosis***

**Fig. 4:**
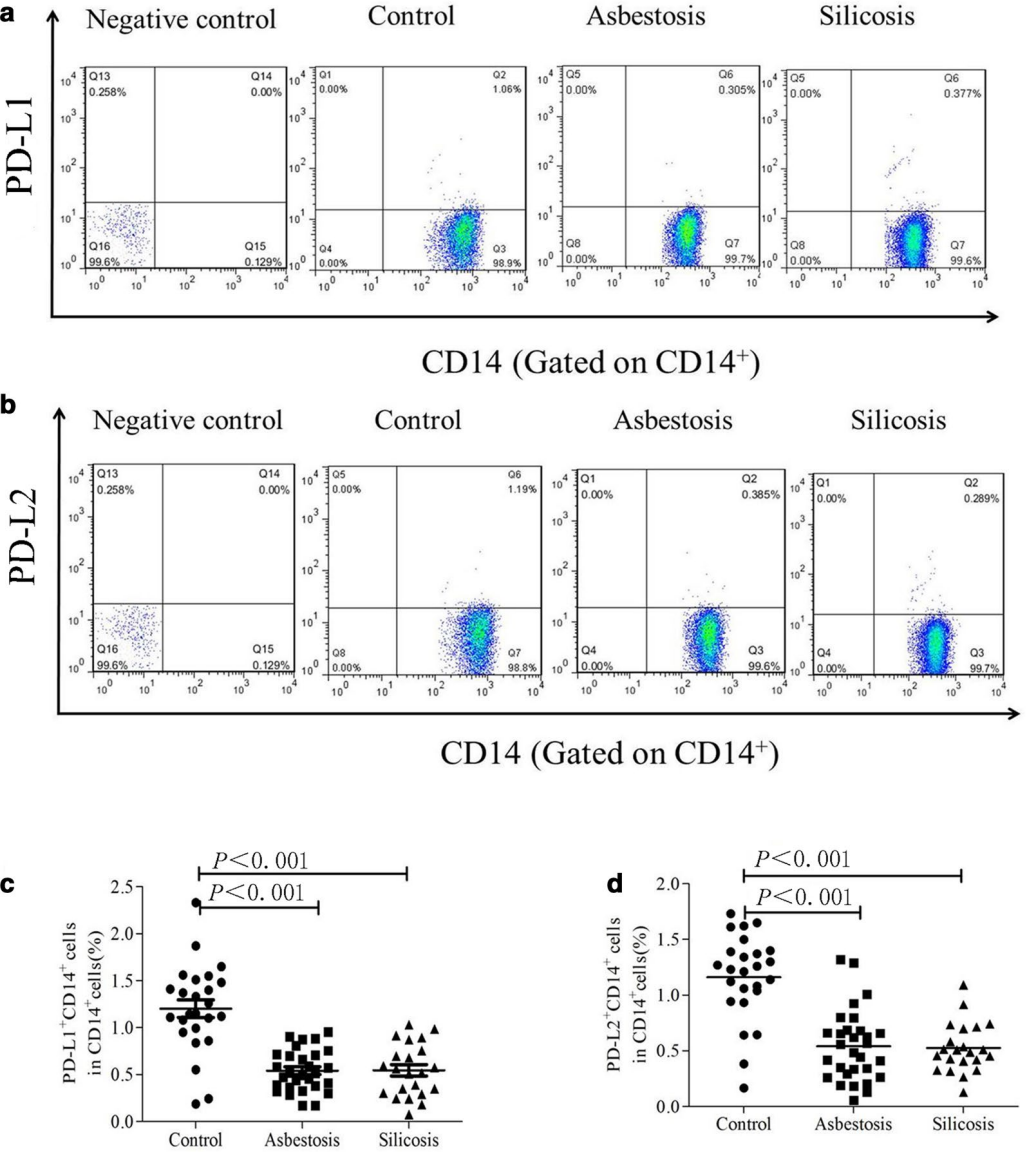
Analysis of PD-L1 or PD-L2 expression on CD14^+^ cells in PB. Representative flow cytometry analysis of the expression of PD-L1 (**a**) or PD-L2 (**b**) on CD14^+^ cells. The percentages of PD-L1^+^CD14^+^ (**c**) or PD-L2^+^CD14^+^ (**d**) cells in healthy controls and patients with asbestosis or silicosis

We further detected PD-L1 (CD274) and PD-L2 (CD273) expression on CD14^+^ monocytes in PB. As illustrated in Fig. 4a, c, the percentages of circulating PD-L1^+^ CD14^+^ monocytes were significantly decreased in the asbestosis (mean 0.541%) and silicosis (mean 0.544%) groups compared to the healthy control group (mean 1.203%, *P* < 0.01). Similarly, lower percentages of circulating PD-L2^+^ CD14^+^ monocytes were observed in the asbestosis (mean 0.541%) and silicosis (mean 0.525%) groups than in the healthy controls (mean 1.161%, *P* < 0.01) (Fig. 4b, d). A significant difference in PD-L expression on monocytes was not observed between the asbestosis and silicosis groups (Fig. 4c, d).

### T cell activation status in patients with asbestosis and silicosis

**Fig. 5:**
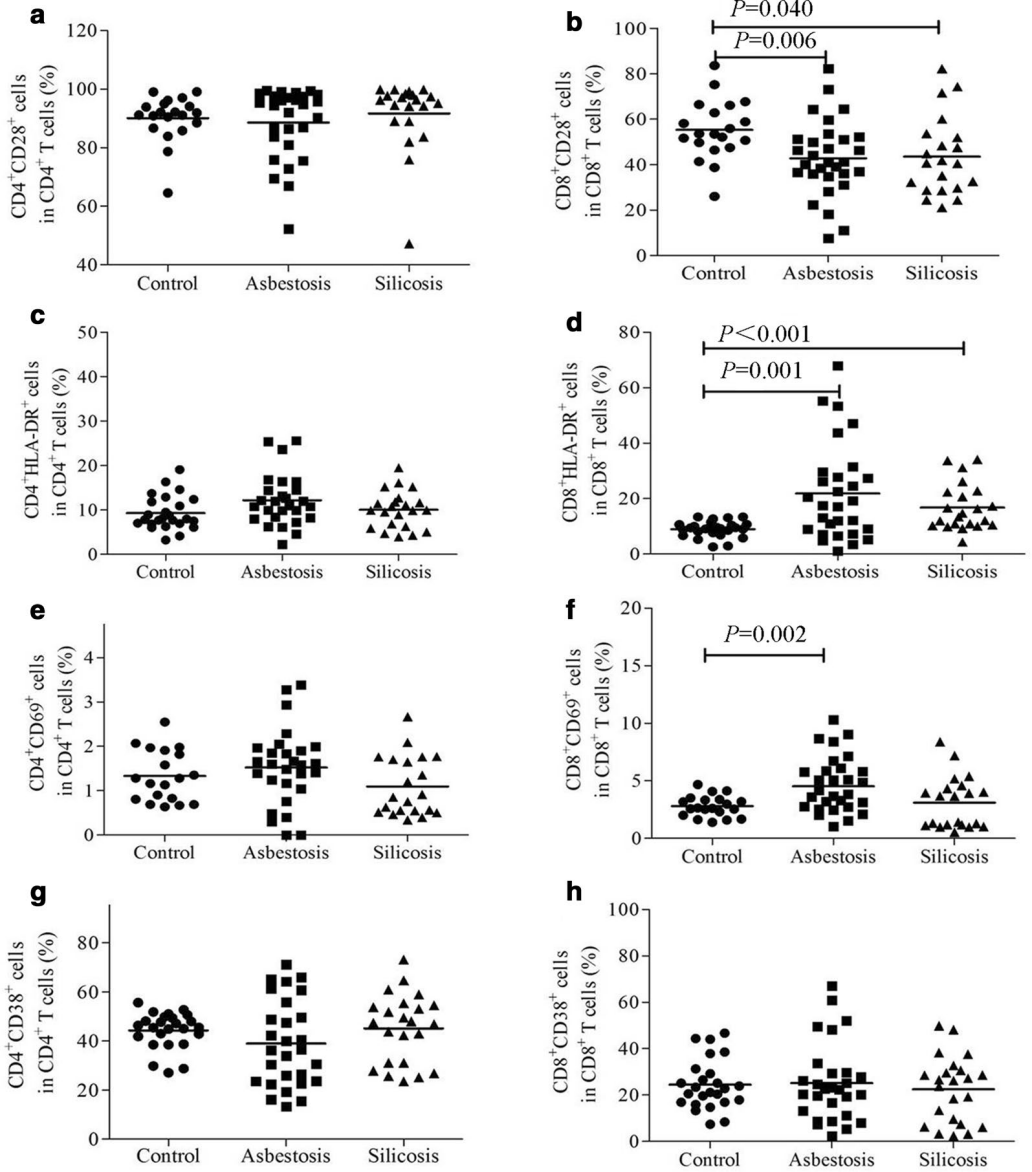
The activation status of effector T cells in PB. The percentage of CD28^+^ CD4^+^ (**a**) or CD28^+^ CD8^+^ (**b**) T cells among the three groups; the percentage of HLA-DR^+^ CD4^+^ (**c**) or HLA-DR^+^CD8^+^ (**d**) T cells among the three groups; the percentage of CD38^+^ CD4^+^ (**e**) or CD38^+^CD8^+^ (**f**) T cells among the three groups; and the percentage of CD69^+^ CD4^+^ (**g**) or CD69^+^CD8^+^ (**h**) T cells among the three groups

**Fig. 6:**
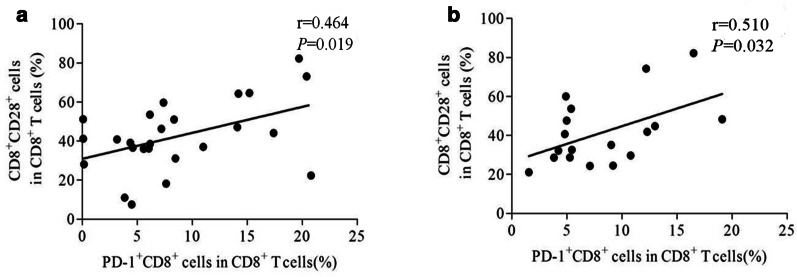
Correlations between PD-1^+^CD8^+^ T cell fractions with CD28^+^CD8^+^ T cell fractions. The PD-1^+^CD8^+^ T cell fraction positively correlated with the CD28^+^CD8^+^ T cell fraction in the PB of patients with asbestosis (r = 0.464, *P* = 0.019) (**a**) or silicosis (r = 0.510, *P* = 0.032) (**b**)

We also explored the activation status of effector T cells in patients with asbestosis and silicosis. The percentages of circulating CD28^+^CD8^+^ T cells were significantly lower in patients with asbestosis and silicosis than in the healthy controls (*P* < 0.05) (Fig. 5b), and the percentages of circulating HLA-DR^+^CD8^+^ T cells were significantly higher in both groups than in the healthy controls (*P* < 0.05) (Fig. 5d). No significant differences were detected in the percentages of CD28^+^CD4^+^ T cells, HLA-DR^+^CD4^+^ T cells and CD69^+^CD4^+^ T cells between the asbestosis and silicosis groups (*P* > 0.05, Fig. 5a, c, and e). The percentage of CD69^+^CD8^+^ T cells in PB was significantly increased in the asbestosis group compared to the healthy control group (*P* < 0.05), and the percentage of CD69^+^CD8^+^ T cells tended to be higher in the silicosis group than in the healthy control group (Fig. 5f). The percentages of CD38^+^ CD4^+^ T cells and CD38^+^ CD8^+^ T cells in PB were not different among the three groups (Fig. 5g–h). Notably, the percentage of PD-1^+^ CD8^+^ T cells was positively correlated with the percentage ofCD28^+^CD8^+^ T cells in the asbestosis (r = 0.464, *P* = 0.019; Fig. 6a) and silicosis groups (r = 0.510, *P* = 0.032; Fig. 6b).

### Downregulated PD-1 expression was correlated with the percentage of FVC predicted

**Fig. 7:**
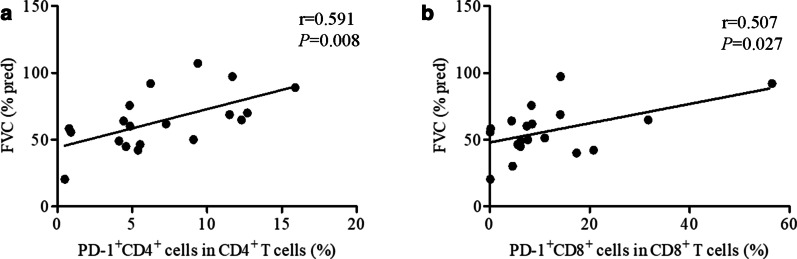
Correlations between PD-1 expression and FVC% predicted in patients with asbestosis. PD-1^+^CD4^+^ T cell fractions (r = 0.591, *P* = 0.008) (**a**) and PD-1^+^CD8^+^ T cell fractions (r = 0.507, *P* = 0.027) (**b**) positively correlated with FVC predicted. FVC, forced vital capacity

Pulmonary functional parameters have been used to predict the severity of chronic fibrotic lung diseases [13]. Here, we assessed the correlations between PD-1 expression and pulmonary function parameters and found that the proportions of PD-1^+^ CD4^+^ T cells (r = 0.591, *P* = 0.008) and PD-1^+^CD8^+^ T cells (r = 0.507, *P* = 0.027) were positively correlated with the percentage of FVC predicted in the asbestosis group (Fig. 7a, b). Furthermore, all patients with asbestosis were classified into stage I, stage II and stage III subgroups based on CT findings. Although no significant difference was observed among the three stages, the mean PD-1 expression on CD4^+^ T cells or CD8^+^ T cells in patients with stage III asbestosis was lower than that in patients with stage I and stage II disease (Additional file 1: Fig. S1). In addition, we investigated the relationships between PD-1 expression and other pulmonary function parameters: PaO_2_ and the composite physiological index (CPI). We did not identify any correlations between PD-1 expression and DLCO% predicted, TLC% predicted, PaO_2_, or CPI (Additional file 2, 3: Figs. S2–S3).

## Discussion

In the current study, we documented significantly decreased PD-1 expression on CD4^+^ T or CD8^+^ T cells in PB from patients with asbestosis and silicosis, and the proportions of CD4^+^/CD8^+^ PD-1^+^ T cells were positively correlated with the percentage of FVC predicted in patients with asbestosis. CD28 expression was significantly decreased on CD8^+^ T cells in PB from patients with asbestosis, while CD69 and HLA-DR expression was significantly increased on CD8^+^ T cells in PB from these patients. In addition, the proportions of CD8^+^ PD-1^+^ T cells were positively correlated with the proportions of CD8^+^ CD28^+^ T cells in the PB of patients with asbestosis.

Asbestosis and silicosis are ILDs characterized by chronic diffuse aseptic lung tissue inflammation caused by the inhalation of asbestos and small silica crystals. These inorganic dusts deposit in the distal airways, where macrophages residing in the small airways are postulated to ingest silica crystals and asbestos, thereby initiating an inflammatory response [14]. During this process, lymphocytes and other cells become activated and begin to secrete profibrotic cytokines and growth factors, such as transforming growth factor (TGF)-β1, interleukin (IL)-13 and platelet-derived growth factor (PDGF) [15]. Monocytes/macrophages, T lymphocytes and associated cytokines play an important role in the development of human fibrotic lung diseases and animal models of pulmonary fibrosis [16]. However, the detailed mechanism of T lymphocyte activation in patients with asbestosis has not been clarified.

T cell activation requires two signals [17]. The first signal is activation of the T cell receptor via the recognition of antigens presented by the major histocompatibility complex on antigen-presenting cells (APCs). The second signal involves the ligation of costimulatory and coinhibitory molecules expressed on APCs and T cells. PD-1 was first isolated from a murine T cell hybridoma undergoing programmed cell death by Ishida and colleagues in 1992 [18]. PD-1, a coinhibitory molecule, is expressed on T cells, Tregs, B cells, activated monocytes, dendritic cells (DCs), natural killer (NK) cells and natural killer T cells [8]. PD-1 binds to two ligands: PD-L1 and PD-L2. The PD-1/PD-Ls pathway in the B7/CD28 family plays a critical role in regulating T cell activation and autoimmune tolerance [19, 20]. The binding of PD-1 to PD-L1 or PD-L2 blocks B and T cell proliferation, inhibits the secretion of cytokines and influences T cell survival [9, 21]. The PD-1/PD-Ls axis not only represents a relevant negative feedback loop for maintaining immune homeostasis but is also of crucial importance for restricting tumour immunity and controlling the inflammatory response to injury in normal lung tissues. Numerous studies have indicated that this pathway exerts an effect on granulomatous diseases [22, 23], chronic infections [24–26], tumours [27], and autoimmune diseases [28, 29]. However, the role of PD-1/PD-Ls in occupational pulmonary disease currently remains elusive.

In our study, we first observed decreased expression of molecules in the PD-1/PD-Ls pathway in patients with asbestosis and silicosis. More importantly, the proportions of CD4^+^ PD-1^+^ T cells and CD8^+^ PD-1^+^ T cells in patients with asbestosis were positively correlated with the percentage of FVC predicted. Thus, we speculated that the decreased expression of components of the PD-1/PD-Ls pathway in patients with asbestosis promoted T cell activation, resulting in increased cytokine secretion. Increased T cell activation and cytokine secretion may aggravate the inflammatory response, ultimately leading to more severe pulmonary fibrosis. Idiopathic pulmonary fibrosis (IPF) is also a chronic fibrotic lung disease. IPF is characterized by the excessive accumulation of extracellular matrix in the interstitial and alveolar spaces, leading to scarring and the destruction of the normal pulmonary epithelium [30]. IPF, a similar disease to cancer, should be considered a neoproliferative disorder of the lung [31]. In a previous study, RNA sequencing identified PD-1 as a significantly downregulated gene in human IPF lung tissue obtained by surgical biopsy [32]. However, a significant increase in the expression of PD-L1 in a subset of invasive human lung fibroblasts isolated from explant lung tissues was detected in a recent study, and an increase in soluble PD-L1 levels in serum was observed in patients with IPF compared with healthy controls in another pilot study [33, 34]. Interestingly, in another study, PD-L1 expression was not increased in the peripheral blood of patients with IPF compared to healthy controls, but PD-1 expression was increased significantly on T lymphocytes in both peripheral blood and lung tissue of patients with IPF [35].

In the present study, PD-1 and PD-L1 expression showed different trends in patients with asbestosis and silicosis compared with patients with IPF. A potential explanation for some of the differences is provided below. Asbestosis and silicosis have different aetiologies than IPF. Asbestosis and silicosis are a group of heterogeneous fibrotic lung diseases that develop through the inhalation of asbestos and small silica crystals. Macrophages, key regulators of fibrosis that reside in the small airways, ingest silica crystals and asbestos and produce numerous profibrotic soluble mediators, chemokines, and matrix metalloproteases, thereby controlling extracellular matrix (ECM) deposition. Monocytes and macrophages function as antigen-presenting cells that send costimulatory and coinhibitory signals to T cells and thus have the ability to promote Th2 responses that induce and activate TGF-β1 in macrophages through an IL-13 and matrix metalloproteinase (MMP)9-dependent mechanism [36, 37]. Although PD-L1 expression on macrophages was not tested in our study, PD-L1 and PD-L2 expression on monocytes was decreased. We speculated that the activity of the PD-1/PD-Ls pathway, which creates an inhibitory signal, was decreased, in turn increasing T cell activation and subsequently potentially promoting Th2 responses and resulting in a fibrotic process. Based on numerous findings, macrophages appear to have distinct roles, exhibiting a predominant phenotype that depends on different stimuli or the microenvironment. The inflammatory M1 and anti-inflammatory M2 phenotypes are considered a potential dynamic spectrum of activation [38–40]. Therefore, we speculate that the PD-1/PD-Ls pathway, which acts on monocytes/macrophages in different phases, may produce different results. Heterogeneity in the stimuli, genetic predisposition and diverse signalling mechanisms that promote profibrotic cell phenotypes may also contribute to the difference in PD-1 expression between patients with asbestosis, silicosis and IPF. Thus, the downregulation of the PD-1/PD-Ls pathway might be a specific phenotype or at least a particular developmental phase of asbestosis and silicosis.

Downregulation of the PD-1/PD-Ls pathway potentially leads to overactivation of T cells. Therefore, we detected the effector T cell activation status in patients with asbestosis and silicosis. Activated T cells were detected in patients with asbestosis and silicosis, and these cells exhibited lower CD28 expression and higher HLA-DR expression. CD28, a marker of early T cell activation, is the second T cell activation signal, while HLA-DR is a marker of late T cell activation [41, 42]. CD28 expression is downregulated in response to chronic T cell activation [43]. Based on these findings, T cell activation was induced by chronic inflammation in patients with asbestosis and silicosis. Interestingly, the percentage of PD-1^+^ CD8^+^ T cells was positively correlated with the percentage of CD28^+^ CD8^+^ T cells in PB from patients with asbestosis and silicosis. Therefore, we hypothesized that lower PD-1 expression on T cells was correlated with greater T cell activation and greater CD28 depletion.

To our knowledge, the present study is the first to explore the PD-1/PD-Ls pathway in occupational pulmonary diseases. We performed a comprehensive analysis of PD-1/PD-Ls expression in PB from patients with asbestosis and silicosis. These findings may facilitate the development of therapies for pulmonary fibrosis. However, this study had some inherent limitations. First, the control population was significantly younger than the asbestosis group, whereas it was not different from the silicosis group. A previous study showed that PD-1 mRNA and protein expression levels increase with ageing [44]. Therefore, we speculated that the cause of the lower PD-1 expression in patients with asbestosis might be the disease itself rather than age. However, because these results from the previous study might be inherent to the cohort studied or irreproducible, we were unable to definitely exclude the effects of age. Therefore, broader/age-adjusted control cohorts are needed in future studies for verification. Second, we detected the expression of molecules in the PD-1/PD-Ls pathway in PB from patients with asbestosis and silicosis but did not detect the expression of molecules in the PD-1/PD-Ls pathway in the lung. In addition, we did not obtain evidence that verified a causal relationship between decreases in lung function and PD-1 expression.

## Conclusions

In conclusion, the data provided further evidence showing that the PD-1/PD-Ls pathway plays a role in the pathogenesis of asbestosis and silicosis. We first showed the downregulation of the PD-1/PD-Ls pathway on cells in PB from patients with asbestosis and silicosis. Importantly, lower PD-1 expression on CD4^+^ T or CD8^+^ T cells in PB was positively correlated with the percentage of FVC predicted, implying a link between pulmonary fibrosis development and downregulation of the PD-1/PD-Ls pathway. Based on the results, the PD-1/PD-Ls pathway potentially represents a therapeutic target.

## Supplementary Information

**Additional file 1. Fig. S1:** Analysis of PD-1 expression patients with different stages of asbestosis. PD-1 expression on CD4+ (a) or CD8+ (b) T cells in patients with different stages of asbestosis.

**Additional file 2. Fig. S2:** Correlations between PD-1 expression with DLCO% predicted and TLC% predicted in patients with asbestosis. Correlations between PD-1+CD4+ T cell fractions and DLCO predicted (r = 0.289, *P* = 0.260) (a), PD-1+CD8+ T cell fractions and DLCO predicted (r = 0.038, *P* = 0.875) (b), PD-1+CD4+ T cell fractions and TLC% predicted (r = 0.152, *P* = 0.535) (c); and PD-1+CD8+ T cell fractions and TLC% predicted (r = 0.156, *P* = 0.512) (d) are shown. DLCO, diffusing capacity of the lung for carbon monoxide; TLC: total lung capacity.

**Additional file 3. Fig. S3:** Correlations between PD-1 expression with PaO2 and CPI in patients with asbestosis. Correlations between PD-1+CD4+ T cell fractions and PaO2 (r = − 0.268, *p* = 0.899) (a), PD-1+CD8+ T cell fractions and PaO2 (r = − 0.072, *P* = 0.733) (b), PD-1+CD4+ T cell fractions and CPI (r = 0.399, *P* = 0.091) (c), and PD-1+CD8+ T cell fractions and CPI (r = 0.213, *P* = 0.380) (d) are shown. CPI: composite physiological index.

## Data Availability

The datasets used and/or analysed during the current study are available from the corresponding author upon reasonable request.

## Abbreviations

PD-1: Programmed cell death protein 1
PD-Ls: Programmed death ligands
PB: Peripheral blood
FVC: Forced vital capacity
ILD: Interstitial lung disease
COPD: Chronic obstructive pulmonary disease
CPI: Composite physiological index
HRCT: Chest high-resolution CT
UIP: Usual interstitial pneumonia
NSIP: Nonspecific interstitial pneumonia
PaO_2_: Arterial partial pressure of oxygen
FEV1: Forced expiratory volume in the first second
TLC: Total lung capacity
DLCO: Diffusing capacity of the lung for carbon monoxide
PBMC: Peripheral blood mononuclear cell
IPF: Idiopathic pulmonary fibrosis
TGF-β1: Transforming growth factor-β1
IL: Interleukin
PDGF: Platelet-derived growth factor
APC: Antigen presenting cells
DCs: Dendritic cells
NK: Natural killer
ITIM: Immunoreceptor tyrosine-based inhibitory motif
ITSM: Immunoreceptor tyrosine-based switch motif
ECM: Extracellular matrix
MMP: Matrix metalloproteinase
TIMP: Tissue inhibitor of metalloproteinase
