# Supplementary figures and images for: Downregulation of the PD-1/PD-Ls pathway in peripheral cells correlates with asbestosis severity
Source: BMC Pulm Med. 2021 May 22;21:175. doi: 10.1186/s12890-021-01531-5 (PMC8141175; doi:10.1186/s12890-021-01531-5)

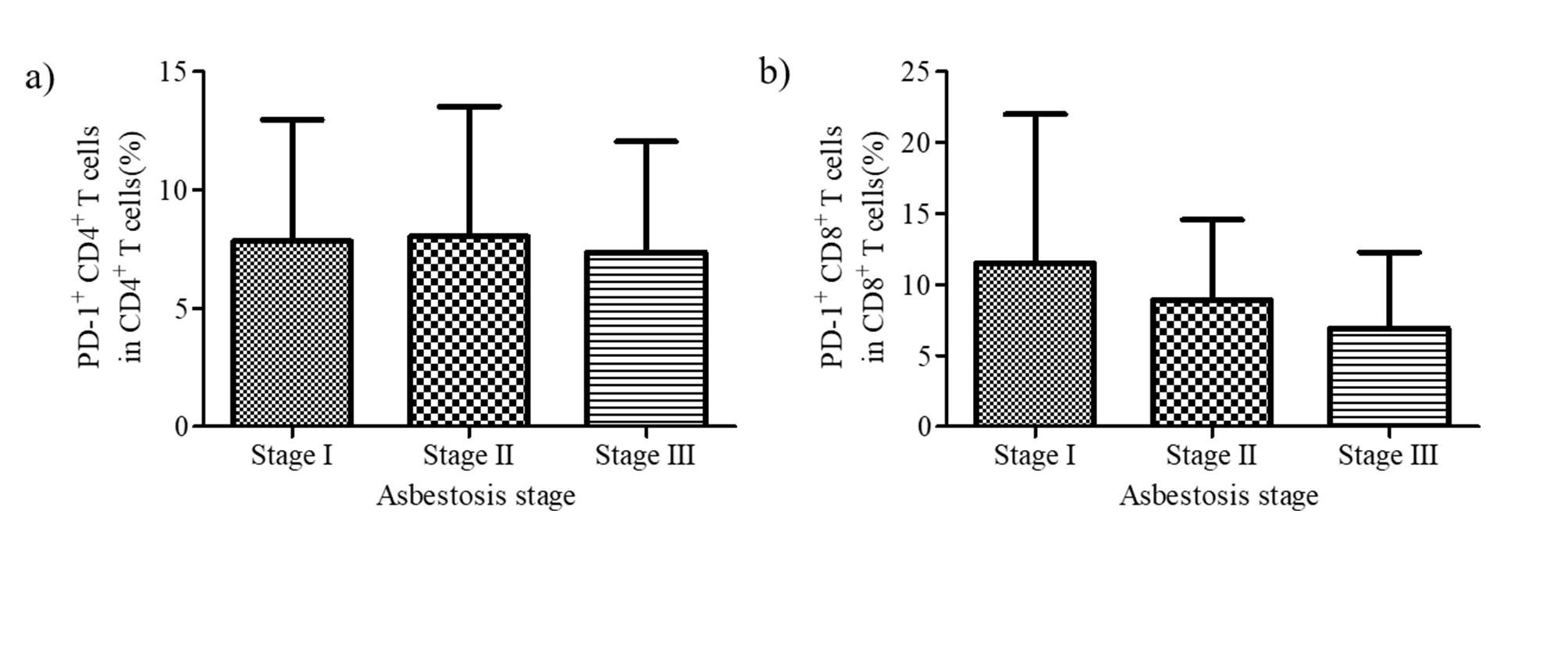

Supplement: Supplementary file 1 — Additional file 1. Fig. S1: Analysis of PD-1 expression patients with different stages of asbestosis. PD-1 expression on CD4+ (a) or CD8+ (b) T cells in patients with different stages of asbestosis. [file 12890_2021_1531_MOESM1_ESM.tif]

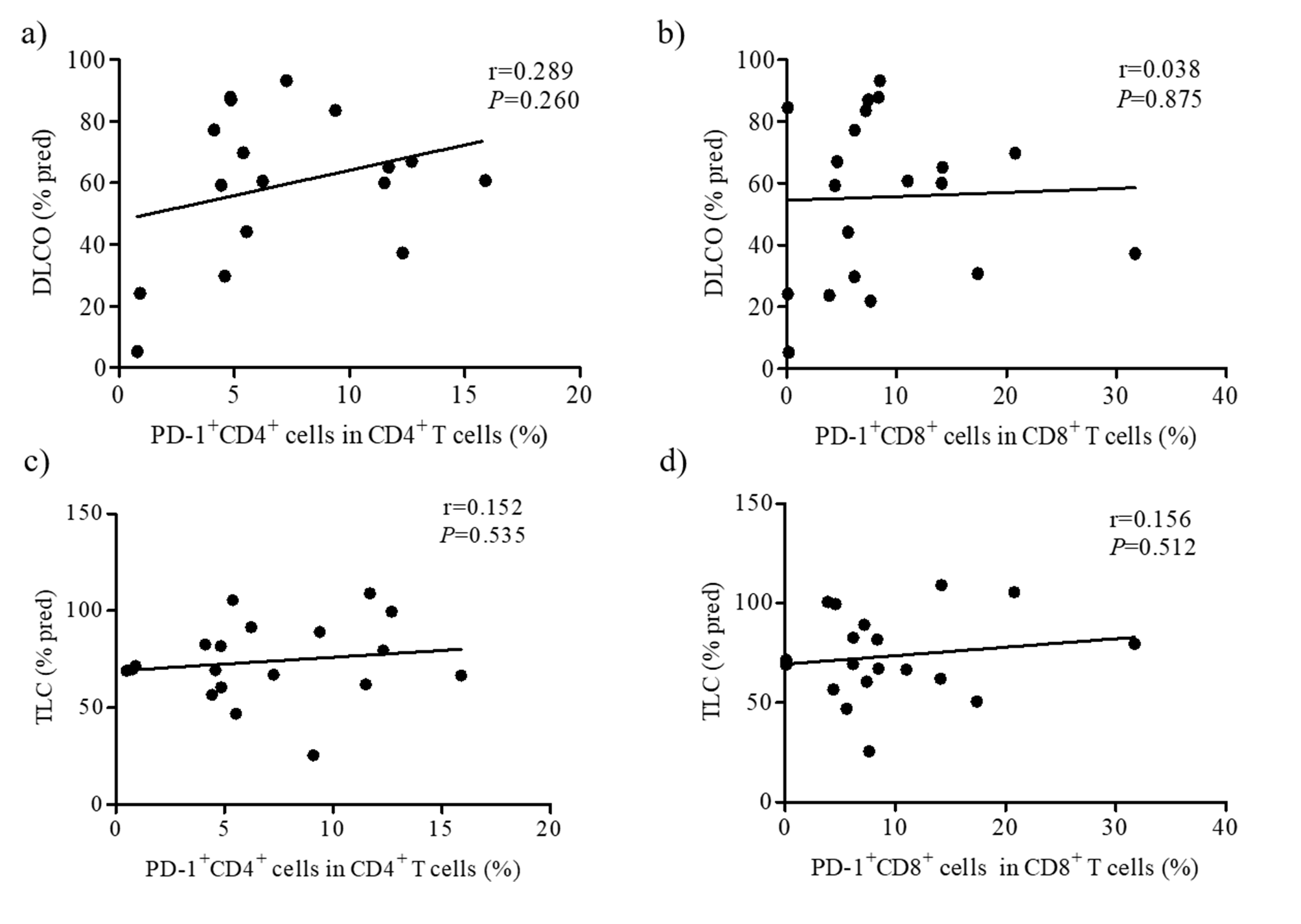

Supplement: Supplementary file 2 — Additional file 2. Fig. S2: Correlations between PD-1 expression with DLCO% predicted and TLC% predicted in patients with asbestosis. Correlations between PD-1+CD4+ T cell fractions and DLCO predicted (r = 0.289, P = 0.260) (a), PD-1+CD8+ T cell fractions and DLCO predicted (r = 0.038, P = 0.875) (b), PD-1+CD4+ T cell fractions and TLC% predicted (r = 0.152, P = 0.535) (c); and PD-1+CD8+ T cell fractions and TLC% predicted (r = 0.156, P = 0.512) (d) are shown. DLCO, diffusing capacity of the lung for carbon monoxide; TLC: total lung capacity. [file 12890_2021_1531_MOESM2_ESM.tif]

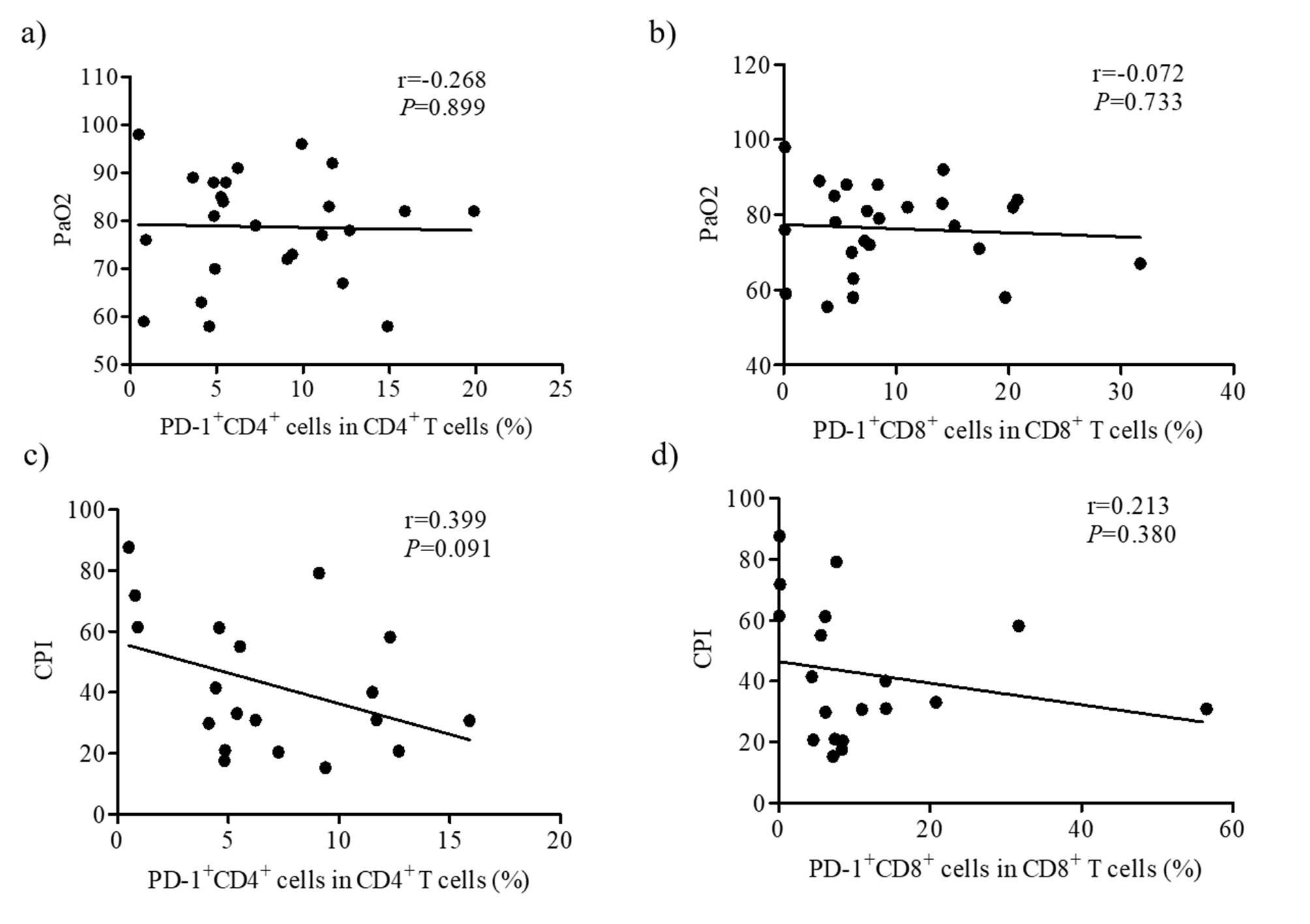

Supplement: Supplementary file 3 — Additional file 3. Fig. S3: Correlations between PD-1 expression with PaO2 and CPI in patients with asbestosis. Correlations between PD-1+CD4+ T cell fractions and PaO2 (r = − 0.268, p = 0.899) (a), PD-1+CD8+ T cell fractions and PaO2 (r = − 0.072, P = 0.733) (b), PD-1+CD4+ T cell fractions and CPI (r = 0.399, P = 0.091) (c), and PD-1+CD8+ T cell fractions and CPI (r = 0.213, P = 0.380) (d) are shown. CPI: composite physiological index. [file 12890_2021_1531_MOESM3_ESM.tif]
